# Discovery and Visualization of the Hidden Relationships among N-Glycosylation, Disulfide Bonds, and Membrane Topology

**DOI:** 10.3390/ijms242216182

**Published:** 2023-11-10

**Authors:** Manthan Desai, Amritpal Singh, David Pham, Syed Rafid Chowdhury, Bingyun Sun

**Affiliations:** 1 Department of Molecular Biology and Biochemistry, Simon Fraser University, Burnaby, BC V5A 1S6, Canada; manthan_desai@sfu.ca; 2 Department of Computing Science, Simon Fraser University, Burnaby, BC V5A 1S6, Canada; aamritpa@sfu.ca (A.S.); ddkpham@gmail.com (D.P.); rafid_haider@sfu.ca (S.R.C.); 3 Department of Chemistry, Simon Fraser University, Burnaby, BC V5A 1S6, Canada

**Keywords:** post-translational modifications, transmembrane domains, N-glycosylation, disulfide bonds, membrane protein topology, endoplasmic reticulum, protein structure and function

## Abstract

Membrane proteins (MPs) are functionally important but structurally complex. In particular, MPs often carry three structural features, i.e., transmembrane domains (TMs), disulfide bonds (SSs), and N-glycosylation (N-GLYCO). All three features have been intensively studied; however, how the three features potentially correlate has been less addressed in the literature. With the growing accuracy from computational prediction, we used publicly available information on SSs and N-GLYCO and analyzed the potential relationships among post-translational modifications (PTMs) and the predicted membrane topology in the human proteome. Our results suggested a very close relationship between SSs and N-GLYCO that behaved similarly, whereas a complementary relation between the TMs and the two PTMs was also revealed, in which the high SS and/or N-GLYCO presence is often accompanied by a low TM occurrence in a protein. Furthermore, the occurrence of SSs and N-GLYCO in a protein heavily relies on the protein length; however, TMs seem not to possess such length dependence. Finally, SSs exhibits larger potential dynamics than N-GLYCO, which is confined by the presence of sequons. The special classes of proteins possessing extreme or unique patterns of the three structural features are comprehensively identified, and their structural features and potential dynamics help to identify their susceptibility to different physiological and pathophysiological insults, which could help drug development and protein engineering.

## 1. Introduction

Membrane proteins (MPs) are a vital component of the cellular proteome. This class of proteins constitutes close to 30% of the proteome and functions in many critical cellular processes, such as solute and ion transport, cell–cell interactions, adhesion, and signal transduction [1]. Therefore, MPs account for more than 60% of drug targets [2] and are frequently used as diagnostic markers for various diseases.

The topology and folding of MPs are key to their functions due to a strong correlation between them [3]. The arrangement of structural elements such as alpha helices, beta strands, and loops across the lipid bilayer heavily influences protein functions such as ion transport and signal transduction. Protein topology and folding can dictate the location of the protein active sites, thereby modulating its accessibility to ligands, ions, and other proteins [4]. Topology can also influence conformational changes that typically occur in MP functional cycles [5]. Most MPs perform their functions ranging from small movements within the transmembrane (TM) regions to large interdomain movements involving multiple other proteins. Therefore, understanding the link between topology and conformations can be key to interpreting protein functions.

The unique properties of membrane topology, folding, expression, and cellular localization render MPs technically challenging to study. The low solubility, low expression, and high PTMs have prevented highly informative X-ray and NMR structural analyses [6,7] of these proteins. The dynamic nature and complex topogenic signals, such as protein–substrate and protein–lipid interactions as well as cell signaling-regulated PTMs, introduce ample variability in topology formation. In addition, translocon dynamics further complicate topology development, because many accessory factors of translocon only transiently or sub-stoichiometrically associate with the core machinery [8]. As a result, polypeptides with identical sequences can span the membrane differently in a concept known as topological heterogeneity [9].

To overcome the challenges in the experimental determination of MP topology and structure, computational algorithms such as machine learning (ML) have been developed to predict these features. Formally, ML is defined as the automated obtaining of patterns and correlations from data using complex but efficient algorithms [10]. In the scope of transmembrane topology, the latest ML models excel in predicting transmembrane protein segments by analyzing sequence, hydrophobicity patterns, and structural features. A major benefit of ML models in this field is their throughput, which allows for the structural interpretation of the entire MP class.

ML models have experienced several generations of evolution, from the early ones developed by Jack Kyte and Rusell Doolittle in 1981 [11] to the dense alignment surface (DAS) method that appeared in 1997 [12] and the support vector machine (SVM) model in the 2000s [13]. The previously best-performing prediction used the hidden Markov model (HMM) developed by Anders Krogh et al., in 2001 [14]. Neural networks were applied to MP structural prediction in the 1990s [15]. With recent developments in both hardware and computing software, neural networks have been revived and have advanced numerous data-driven research fields. Krogh et al., recently applied deep learning in a neural network to optimize TMHMM and resolved Deep TMHMM, which achieved the highest accuracy in the prediction of TM protein topology so far [16] and recognizes signal peptides and beta-barrels in MPs. It is foreseeable that the future structural challenges in MPs will lie in the discovery of their dynamics (i.e., conformational changes) when interacting with their binding partners and/or upon environmental insults.

In addition to TMs, growing evidence has pinpointed that PTMs are critical in maintaining membrane protein topology and structure. For example, via proteolytic cleavage or the addition of structural- or charge-modifying groups to one or more residues, the topology of MPs can be altered [17]. The most common PTMs in MPs are disulfide bridges (SSs) and N-linked glycosylation (N-GLYCO) bonds. The former forms between thiol groups in two cysteine residues intramolecularly or intermolecularly, and the latter typically forms on sequons [17] even though atypical N-GLYCO is frequently observed [18]. Both PTMs have compartment specificity. Their location can be used to determine the topology of MPs [19].

With the large amount of structural information available at the human proteome level, we performed a series of statistical analyses with a particular focus on SSs and N-GLYCO acquired from the UniProt database in conjunction with membrane topology predicted by Deep TMHMM. The reason to choose these three structural features for analysis is because all of them are chiefly formed in the ER. Membrane insertion of TMs mainly depends on the lateral exit through translocon in the ER membrane [8], which also complexes with the N-GLYCO machinery—oligosaccharyl transferase (OST) [20]. An important paralog to the catalytic enzyme STT3A in OST is STT3B, which has oxidoreductase activity that can form mixed disulfide bonds with target proteins through its components MAGT1 or TUSC3 [20,21]. In addition, a number of ER-resident glycoenzymes and glycochaperons also form complexes with thiol-disulfide oxidoreductases or carry oxidoreductive activity themselves [22,23]. We therefore hypothesize that a close relationship may exist among these structural features.

The analyses allowed us to discover several hidden relationships among SSs, N-glycans, and TMs, including the correlation between their occurrence with protein length and their relative location in a protein. Functional enrichment analyses further revealed important protein classes that possess these structural features, such as the GPCRs, ion channels, adhesion molecules, enzymes, and receptors functioning in signaling and immune responses. In addition, using the compartmental specificity in SSs and N-GLYCO, we identified a set of proteins with conflicting topology from topology prediction. These conflicts allowed us to discover the potential dynamics of PTMs. To allow other researchers to visualize these hidden correlations, we developed a display portal for the proteins showing extreme TMs, PTMs, and conflicts. We further provided supplemental lists on the proteins possessing special TMs and PTMs. The obtained information helps to elucidate how the complex topology and structures form and evolve from the primary sequence of proteins. Such global information can support a better functional understanding of MPs and help a potential prediction of the susceptibility of selective MP classes to particular environmental and physiological insults. The implications for structural flexibility and constraints in MPs can also facilitate drug design and protein engineering [24,25,26].

## 2. Results and Discussion

### 2.1. Length and Count Distribution

**Figure 1:**
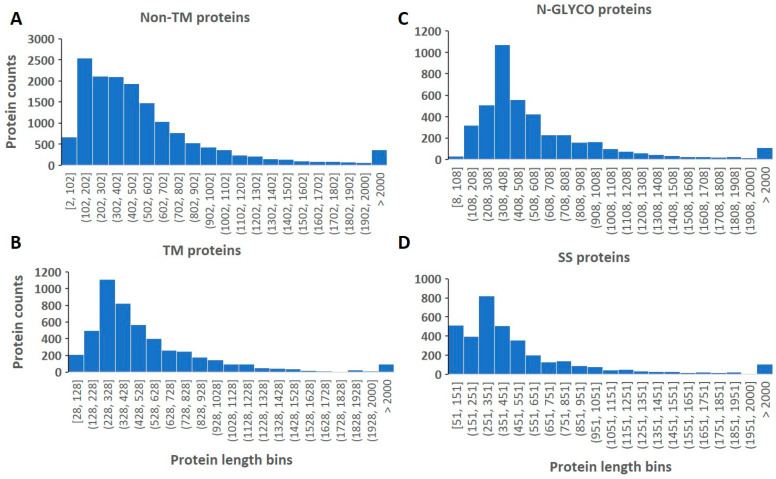
The length distribution of proteins in non-TM (**A**), TM (**B**), N-GLYCO (**C**), and SS (**D**) proteomes.

The examination of the length distribution in various features shows some interesting properties. The length distribution of the human proteome segregated by the TMs predicted by Deep TMHMM is shown in Figure 1A,B. Clearly, the proteins with TMs possess different length distributions than those without TMs. The non-TM proteins peak at a shorter length of 102–202 amino acids than that of TM proteins, whose length peaks at 228–328 amino acids. Otherwise, the average protein lengths in the non-TM proteome and TM proteome are very similar, with TM proteins having 558.42 (N = 4947) and non-TM proteins having 557.83 (N = 15,464) amino acids.

The length distribution in the N-GLYCO and SS proteomes was also analyzed and is shown in Figure 1C,D, respectively. Differences were also observed between these two PTMs, with N-GLYCO preferring longer proteins over SS. The respective peak lengths for the N-GLYCO proteome and SS proteome are 308–408 (N = 4214) and 251–351 (N = 3588) amino acids. The overall mean length of the two proteomes is also similar, with the N-GLYCO proteome of 611.93 amino acids and the SS proteome of 559.69 amino acids. The mean lengths in the two PTM proteomes are close to those of TM and non-TM proteomes; however, the peak distributions among these four proteomes are different. Therefore, the length distribution is a more sensitive analysis in disclosing the hidden differences among these large proteomes.

**Figure 2:**
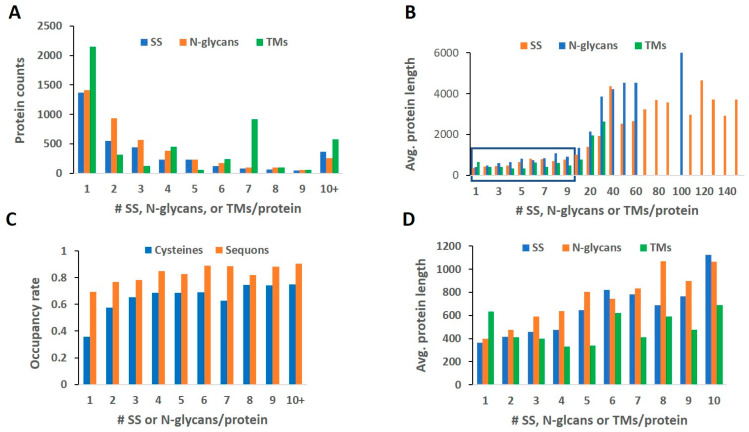
Distribution of protein counts, average protein length, and occupancy rates as a function of feature occurrence in one protein. (**A**) Protein counts vs. numbers of SSs, N-glycans, or TMs/protein; (**B**) Average protein length vs. numbers of SS, N-glycans, or TMs/protein; (**C**) Occupancy rates of cysteines and sequons as a function of the numbers of SS and N-glycans/protein; (**D**) A zoom-in view of the boxed area in (**B**).

After length analysis, we examined the frequency of TMs, SSs, and N-glycans in each protein of their corresponding proteomes. Figure 2A summarizes the results. Among all three structural features, the single occurrence had the dominant population in all proteomes. In contrast, some proteins possess an extremely high occurrence of a single feature. The highest number of TMs in a single protein is 38, carried by Piezo-type mechanosensitive ion channel component 1 (or membrane protein induced by beta-amyloid treatment). The highest SS bond in a protein is 159, carried by Prolow-density lipoprotein receptor-related protein 1 (LRP-1); for N-GLYCO, it is 102, carried by Mucin-16 (or ovarian cancer-related tumor marker CA125, ovarian carcinoma antigen CA125). Some of these extreme cases, such as Mucin-16, would skew our later analyses and have been left out as to be mentioned below.

Overall, the occurrence of SSs and N-glycans showed a clear decrease in protein counts when the multiplication of these features increased in a protein but not in TMs. For TMs, the protein populations carrying 1 and 7 TMs show large spikes; otherwise, the distribution of other TM counts in proteins seems random. The large population of MPs carrying seven TMs clearly indicates the specialty of this particular class of MPs. A further functional enrichment analysis indicated that the majority of the proteins are G protein-coupled receptors (GPCRs), specifically olfactory receptors, which have the largest population of GPCRs and are poorly studied due to their extremely low abundance. GPCRs are known to mediate most cellular responses to hormones and neurotransmitters [27]. The malfunction of GPCRs can cause serious disorders such as heart failure, stroke, and cancer; hence, they are extensively targeted via drugs. As of 2017, 134 GPCRs are targets for an estimated 700 approved drugs in the US or the EU [28].

As discovered above, proteins carrying these features tend to peak at a relatively longer length than otherwise, and we wanted to evaluate whether the length also correlates with the frequency of occurrence of these features. Figure 2B shows the average protein length as a function of feature multiplication/protein in each respective proteome. In the cases of SSs and N-GLYCO, the protein length shows a positive trend for the modification frequency up to 30 counts/protein. When above this cutoff, the protein length seems to stagger except for Mucin-16 (only one incidence in the N-GLYCO proteome contributed to the 110–100 bin). An investigation of the high-multiplication proteins (counts > 30) shows that the features are typically packed in dense repeats, such as EGF and CUB repeats, which have minimized the demand in protein length. Therefore, the proteins with high multiplication features can be cataloged into special classes that also possess unique functions to be analyzed later in the Section 3.5.

When zooming in to the low multiplication region with counts fewer than 10, as shown in Figure 2D, TMs do not follow the length correlation exhibited by SSs and N-glycans. The shortest mean length was found in those carrying four and five TMs, rather than single-TM carriers. This unique behavior might originate from the working principle of membrane insertion chiefly via the translocon that functions co-translationally with primarily linear amino acid chains that emerge from ribosomes [8]. Therefore, a high number of TMs can be stacked densely within a small span connected only by short loops [8]. It is also recognized that helical hairpins can form in closely spaced TMs after ribosomes [29] and insert into the membrane together instead of sequentially. Both computational simulations and experimental evaluation show that TM oligomerization in the membrane is sequence-dependent and energetically favored [30]. Therefore, in many cases no additional enzymes are necessary in the insertion processes. Furthermore, a novel theory also emerged to view TM oligomerization from the local concentration perspective [31], in which membrane tethering by TMs reduced the 3D free localization space and confined the TMs in 2D to increase local contact for oligomerization. Helix–helix TM interactions are known to occur in both low-TM and high-TM proteins, such as ion channels. Sodium ion channels with 24 TMs [32] can form helix bundles in lipids. The hydrophobic side chains in the bundle face the lipid bilayer, while the hydrophilic residues face towards the center; in such a hydrophobic lipid bilayer, they are pierced through by a hydrophilic pore formed by channel proteins to allow charged ions to pass [32]. In contrast, the formation of N-GLYCO and SSs requires enzymatic catalysis, in which the multiplication of these PTMs in a protein occurs sequentially, and each reaction requires a specific local conformation for recognition and release [33,34]. Except for the high-density repeats, most low-occurrence multiplications for SSs and N-glycans do not possess the synergistic aggregational force that exists in TMs. The multiplication of SSs and N-glycans behaved more additively, which could explain the observed length dependence in most low-multiplication proteins. In summary, we observed a similar behavior of SSs and N-glycans that is distinct from TMs in their corresponding proteomes.

### 2.2. Rate of Modification

Because the occurrence of SSs is based on the presence of cysteine residues, and N-GLYCO mainly takes place in sequons, we further analyzed the utilization rate of cysteines and sequons in the SS proteome and N-glycoproteome, as shown in Figure 2C. Overall, the sequon utilization rate is higher than that of cysteines. A stable modification rate of sequons was observed regardless of N-glycan occurrence in the N-GLYCO proteome. However, in the low SS proteins (1–2 counts/protein), a drop in the cysteine utilization rate was observed. In both the N-GLYCO and SS proteomes, a relatively high utilization rate (>50%) was observed, suggesting that the presence of sequons and cysteines in the protein sequence is not random. Among all amino acids, cysteine has the least abundance in proteins [35], and its presence is often conserved and functionally important [36]. SS formation is a reversible reaction [37], and free cysteines in proteins increase the risk of SS scrambling in their folding [38,39] and function during environmental insults such as heat shock [40] and oxidative stresses [37]. Our observation prompted us to further analyze how these features relate to each other in individual proteins.

### 2.3. Hierarchical Clustering

Because SSs and N-glycans are common structural features on MPs, we wanted to further explore whether hidden relationships exist between the two PTMs and TMs in individual proteins at the proteome level. In the past, we observed a complementary relationship between TMs and N-glycans from our experimentally obtained N-glycoproteome [41], and we also discovered a complex relationship between SSs and N-glycans [22] in the literature. Here, we wanted to see whether SSs would behave similarly to the N-glycans in the complementary relation to TMs. To study this, we focused on the SS/N-GLYCO proteome (N = 2722).

**Figure 3:**
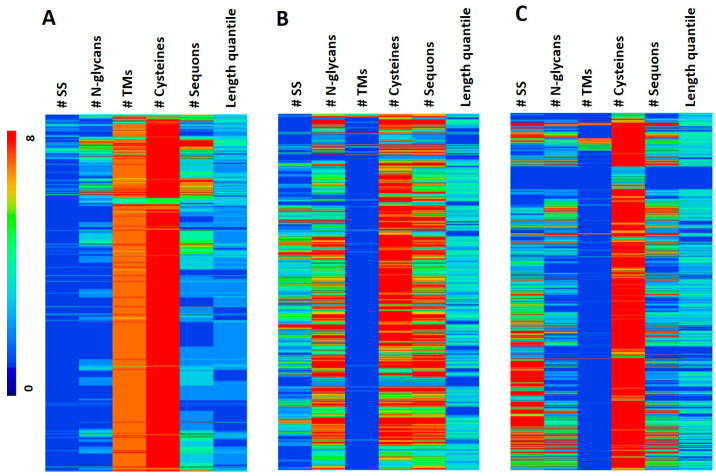
The heatmap of the three clusters of the SS/N-GLYCO proteome based on the counts of SSs, N-glycans, TMs, cysteines, and sequons, as well as the length quantile of the corresponding protein. Length quantile refers to dividing the length of each protein into four equal parts from N-terminus to C-terminus, and their corresponding value is 1–4. (**A**) Heatmap of Cluster 1; (**B**) Heatmap of Cluster 2; (**C**) Heatmap of Cluster 3. Each row represents one protein in the respective proteome, and the color encodes the count values of each protein for a specific property described by each column. The correspondence between different colors and the values is provided as the sidebar on the left.

To evaluate the overall properties in the SS/N-GLYCO proteome, we first performed hierarchical clustering. The results showed three main clusters. The distinct characteristics in Cluster 1 (Figure 3A) from the remaining two clusters are the high TM, low SS, and low N-glycan counts. The remaining two clusters are close to each other, with Cluster 2 (Figure 3B) having relatively high sequon and N-glycan counts and Cluster 3 (Figure 3C) having high cysteine and SS counts. Both Clusters 2 and 3 are low on TMs. Complementarity in TM to the two PTMs on proteins becomes obvious to us, in which TMs are not only complementary to N-GLYCO but also to SSs.

**Figure 4:**
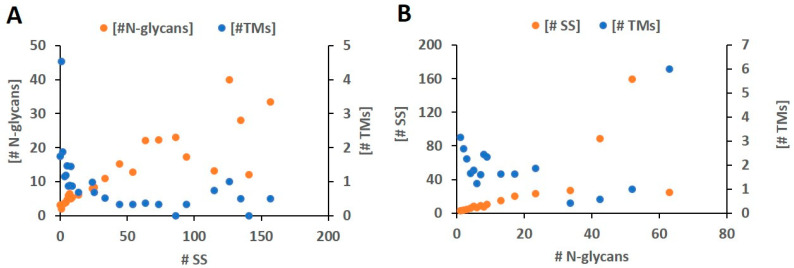
Scatter plot of the numbers of N-glycans, TMs, and SSs in a protein. (**A**) The average numbers of N-glycans and TMs as a function of SS counts in a protein; (**B**) The average numbers of SSs and TMs as a function of N-glycan counts in a protein.

To further confirm this relationship, we used the SS count as the X-axis and examined the average numbers of N-glycans and TMs in the proteins within each SS count bin. The results are shown in Figure 4A. When the SS occurrence per protein is less than 70, consistent with the analysis so far, the N-glycan occurrence exhibits a tight positive linear correlation with SS frequency, whereas the TMs show a negative exponential correlation with the counts of SSs in proteins. The less obvious trends in high multiplications of SSs can be caused by fewer proteins and less statistical power. When using the number of N-glycans as the X-axis and repeating the same analysis, the results shown in Figure 4B are similar to those of Figure 4A. These results suggest that SSs and N-glycans behave similarly. This observation agrees with our previous findings on experimentally examined individual proteins with both SSs and N-GLYCO [22], in which a close but complex relationship was discovered and was supported by the intertwined biogenesis and processing pathways of both PTMs in the ER [23]. We have reviewed in detail how the potential interacting molecular regulations on SSs and N-glycans in the ER result in complex (can be promoting or inhibiting) relationships of both PTMs in individual membranes and secreted proteins [22]. Here, we observed similar behavior of SSs and N-GLYCO at the proteome level.

The negative correlation of TMs with either SSs or N-GLYCO in low-SS or low-N-glycan occurrence proteins agrees with the observation of non-length dependence of TMs in MPs, as shown in Figure 2 and the cluster results in Figure 3. This also agreed with our previous discovery based on the experimentally detected N-GLYCO on membrane proteins [41] using our glyco-peptide capture technique [42]. Such complementarity between TMs and PTMs can be understood from their structural functions. Both SSs and N-glycans help form and stabilize desired conformations of proteins, which can be critical in structurally disordered large loops. When TMs are rich, large, and unstructured, external loops are less frequent than when TMs are poor.

**Figure 5:**
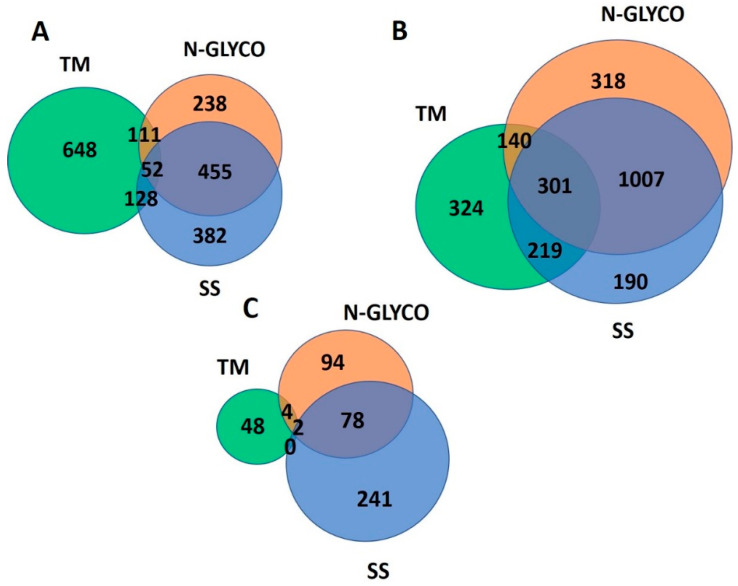
Venn diagrams of proteins carrying various numbers of TMs, N-glycans, and SSs in the SS/N-GLYCO proteome. (**A**) Venn diagram of proteins carrying a single count of either TM, N-glycan, or SS; (**B**) Venn diagram of proteins carrying up to three counts of either TMs, N-glycans, and SSs; (**C**) Venn diagram of proteins carrying more than ten counts of either TMs, N-glycans, and SSs.

This triggered us to further investigate the single-feature carrying proteins that would defy such complementarity. Figure 5A shows the Venn diagram of such comparisons. The smallest section is observed in proteins carrying all features, i.e., 52 entries (2.59% of the total unique entries). The marginal overlap suggests that the complementarity we observed is quite robust with only limited exceptions. To verify whether the remaining entries, particularly those unique to each structural feature proteome, are proteins carrying a large number of complementary structural features, we enlarged the comparison by including the feature occurrence from one to three, as shown in Figure 5B. The size of the section shared by the three features is expanded to 301 entries, which represents an over four-fold increase from 52 entries in Figure 5A. However, the percentage is still marginal, accounting for only 12.1% of the total unique entries. This result suggests that most proteins possess more than three of the other structural feature elements, which explains why MP proteins are challenging to study structurally.

We then shifted the comparison to the higher end of the feature occurrence entries (>10 features/protein), which could also defy the complementarity we observed. The results shown in Figure 5C resemble those of the single-feature comparison shown in Figure 5A. There are only two proteins in the same family shared by all three categories, i.e., NPC intracellular cholesterol transporter 1 (Niemann–Pick C1 protein) and NPC1-like intracellular cholesterol transporter 1 (Niemann–Pick C1-like protein 1), with a cellular localization in endosomes and lysosomes. Both proteins function in transporting low-density lipoproteins and egress cholesterol from endosomes and lysosomes. The two proteins both carry 15 SSs, 19 N-glycans, and 13 TMs. Malfunction of these proteins can cause the over accumulation of cholesterol and glycosphingolipids in the late endosomal/lysosomal compartments. Otherwise, there are only four entries carrying both >10 TMs and >10 N-glycans and 78 entries carrying both >10 N-glycans and >10 SS. No entries carry exclusively both >10 TM and >10 SS. The Venn diagram shown in Figure 5 agrees with the correlation results shown in Figure 4. The small percentage of proteins sharing a high occurrence of the three features (Figure 5C) suggests a certain level of exclusiveness or crowdedness, potentially due to space constraints or avoiding unnecessary redundancy. Accordingly, the large percentage of unique proteins in high-feature-count categories in Figure 5C (TMs at 10.4%, N-GLYCO at 38.2%, and SSs at 52.1%) suggests that MPs of extremely high (>10 counts) structural features are typically high in only one feature. All the proteins in each Venn diagram in Figure 5 are provided in Supplementary Tables S1–S3.

### 2.4. Functional Analysis

The complementary relationship between N-GLYCO, SSs, and TMs revealed in our results inspired us to further disclose the functions carried by these unique classes of proteins in Figure 5. Functional enrichment analysis on the 111 entries having one TM and one N-glycan in Figure 5A revealed proteins in MHC class II, while the 128 entries in Figure 5A having one TM and one SS were enriched by proteins functioning in heparin and heparan sulfate proteoglycan biosynthesis and in the regulation of T cell proliferation. The enrichment analysis was also carried out on the results from the three-feature carrier protein comparisons shown in Figure 5B. The entries shared by one to three TMs and one to three N-glycans are 140 in Figure 5B and enriched by proteins functioning in O-glycosylation, and the entries shared by one to three TMs and one to three SSs exclusively are 219 and are enriched by cell adhesion and cytokine-mediated signal transduction. The commonality between all three categories with features of one to three have 301 entries in Figure 5B and are enriched in immune response proteins. For the largest sharing category, i.e., between one to three N-glycans and one to three SS, there are 1007 entries, which are dominated again by GPCRs. Similarly, in Figure 5A, the section shared by one N-glycan and one SS has 455 entries, the largest overlap section, and is also dominated by GPCRs. The top 10 enriched GO terms from the major sections in Figure 5 are provided in Supplementary Table S4.

The presence of TM domains confined the location of the target protein, and the high number of TMs in low occurrence N-glycan and SS proteins, as we suggested in our previous study, provides stability to the overall protein structure and docking to a special/stable membrane location. Because the membrane is commonly considered a two-dimensional fluidic environment, MPs form close interactions with nearby lipid molecules such that a heterogeneous distribution of lipid molecules in the membrane is observed. Conversely, the phase separation that occurs in the mixed lipid system also encourages the segregation of MPs into different membrane domains to form lipid rafts [43]. The number of TMs is one of the biggest factors in determining the interaction strength of lipids and MPs [30], and the high TMs in low SS and N-GLYCO proteins suggest their special membrane location. Using GPCRs as an example, which have seven TMs and important signaling proteins, they are known to interact closely with lipid components and preferentially localize in lipid rafts and caveolae [44]. Our proteomic level analysis suggests its uniqueness in the entire MPs that is likely contributed to by its seven TMs. A recent discovery suggested that hydrophobic proteins drove phase separation in lipids [45], and GPCRs have been proposed to be one class of these drivers [44]. In addition, the sequon and cysteine presence in high TM proteins, such as GPCRs, is also low, as the utilization ratio is more or less constant across protein populations, as shown in Figure 2C. This result suggests that the high frequency of these PTMs may hinder TM stacking possibly by forming rigid and stable structures in the loop. Through evolution, the potential for these PTMs to occur can be minimized by eliminating their carrier amino acids from the sequence through random mutation and natural selection [46].

### 2.5. Length Quantile Distribution of Features

**Figure 6:**
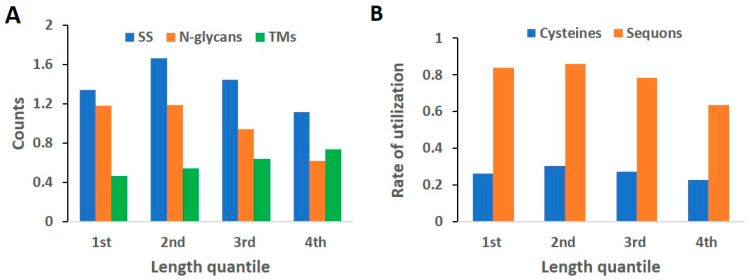
Analysis of feature counts and rate of utilization in length quantile distribution of individual proteins. Length quantile refers to dividing the length of each protein into four equal parts from N-terminus to C-terminus, and named first to fourth quantile. (**A**) Counts of SSs, N-glycans, or TMs as a function of the protein length quantile; (**B**) The rate of utilization of cysteine thiol in SSs and the sequons in N-GLYCO as a function of protein length quantile.

To further examine the relative locations of these features in individual proteins, we analyzed the quantile distribution of these features with respect to individual protein length, as shown in Figure 6. Among the four quantiles, the first quantile describes the N-termini, the last quantile describes the C-termini, and the rest describe the middle section of a protein. Figure 6A shows that both SSs and N-glycans follow a similar trend, in which the N-terminus is more utilized than the C-terminus for the two PTMs. Among the two middle quantiles, the second quantile is used more than the third quantile. Because both PTMs are preferentially compartmentalized in the lumen or the ectodomain of the MPs, the dominance in the N-terminus of MPs suggests a dominant N-terminal orientation, which describes type I MPs; vice versa, the C-terminal SS and N-glycan modification suggests the property of type II MPs. The formation of type II MPs mostly requires co-translational inversion [47] and is not as frequent as that of type I MPs. Similar to previous observations, the trend of TM distribution is opposite to those of N-glycans and SSs. TMs exhibit an increase with increasing quantile, which is from the N-terminus to the C-terminus. Consequently, the complementarity between TMs and PTMs in MPs is reflected in the protein length-quantile distribution as well. We also analyzed the cysteine and sequon quantile distribution and their rate of utilization, as shown in Figure 6B. For both cysteines and sequons, the quantile distribution resembled the SS and N-glycan distributions, respectively, suggesting that the utilization rate is not affected by the protein length.

### 2.6. Conflicts

Conflict analysis was carried out to identify the ectodomain localization of SSs and N-glycans with respect to the MP topology predicted by Deep TMHMM. Compared to an older version of TMHMM 2.0 (364 entries with conflicts), the latest Deep TMHMM prediction (83 entries with conflicts, Supplementary Table S5) has corrected a large number of previous conflicts using the neural network deep learning algorithms, a result suggesting the high accuracy of the current Deep TMHMM prediction. Using the Deep TMHMM results, we found only 1.7% of the total predicted MPs with conflicts, which is within the estimated error rate of Deep TMHMM. This high accuracy rate encouraged us to use the predicted topology to examine the potential location conflicts of sequons and cysteines as well.

**Figure 7:**
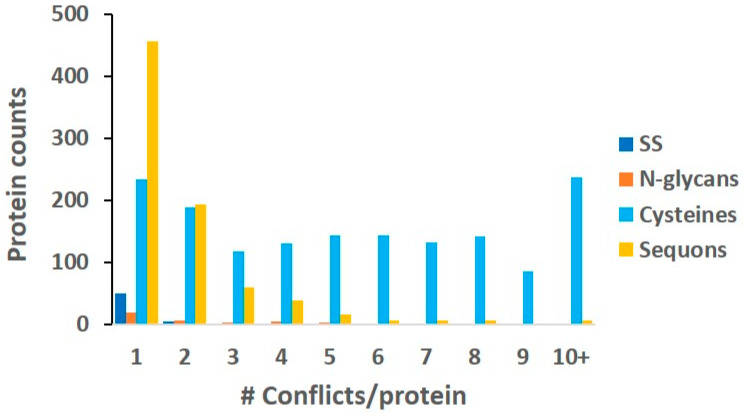
The protein counts as a function of number of conflicts in a single protein. Colors code different types of conflicts. Conflicts were defined as the positions of SSs, N-glycans, or their related cysteines and sequons contradicting the predicted membrane topology of the protein.

Figure 7 summarizes all the results. The protein counts carrying the conflicts are plotted as a function of the conflict occurrence in one protein. The plot includes both the cysteine and sequon conflicts to the Deep TMHMM topology as well as the conflicts of the observed SSs and N-glycans. The SS and N-glycan conflicts are marginal, and most of them only have a single conflict, which are summarized in Supplementary Table S5. What surprised us from the results was the conflict distribution of cysteines and sequons. Sequon conflicts quickly dropped with increasing occurrence, but cysteine conflicts did not, which remained the same. The trend in sequons suggests a nonrandom appearance in the protein sequence because of the agreement with the topology distribution, with single conflict occurring the most. This observation is also congruent with the high utilization rate for N-GLYCO (~85%), as shown in Figure 2B. Experiments using molecular engineering to introduce novel sequons into proteins [46] and clinical analysis of the emergence of novel sequons from genetic mutations [48] have both observed a gain of N-GLYCO that has largely disrupted the normal structure and functions of the target proteins. Collectively, the sequons in proteins do not appear to be random, and most of these sequons have the potential to be N-glycosylated, as Apweiler et al. pointed out 20 years ago when they analyzed the sequons in UniProt [49]. Therefore, we believe that the sequon location defines the dynamic range of this PTM, which could likely trigger local conformational rearrangement due to mostly the observed single conflict. Then, the prevalent cysteine conflicts irrespective of the MP topology suggest that SSs potentially possess larger dynamics than N-GLYCO. Although not all cysteines participate in the SSs, the presence of cysteines does provide the molecular foundation to potentially form SSs. The observed free cysteines can be constrained by local folding [50,51], which could be subject to dynamic perturbations such as oxidative stress and genetic mutations. It is reasonable to speculate that under environmental, developmental, and genetic insults, free cysteines in a protein can increase the possible dynamics of SSs. Collectively, compared to the prevalence of cysteine residues, our results suggest that N-GLYCO (macroheterogeneity) possesses fewer dynamics as SS modifications.

### 2.7. Visualization

**Figure 8:**
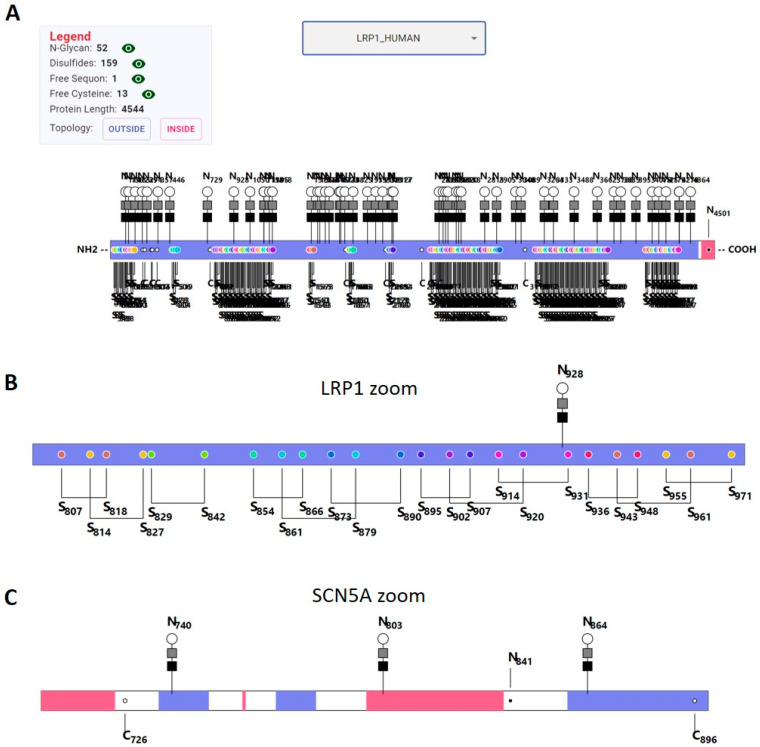
The example displays of the Protein Visualizer. (**A**) The main display of LRP1; (**B**) The zoom display of LRP1 from 800–972 amino acids; (**C**) The zoom display of an N-GLYCO conflict region (N803) in SCN5A between the known compartmentalization of N-glycans to the predicted topology, and the positions of unoccupied sequons and cysteines.

Due to the observed complex relationships, we wanted to visualize these structural features in individual proteins. The existing web applications do not include all the information we study. For example, Protter [52], developed by Dr. Wollscheid’s lab, does not include SS linkages, and the PTM features displayed on the UniProt website do not have TM information. To meet our needs, we developed a graphical user interface (GUI) using Javascript (https://sfu-sun-lab.github.io/protein-visualizer/, accessed on 1 November 2023) and displayed eight properties for every protein. Unique to our visualization, it includes cysteine and sequon positions. Figure 8 shows examples of our GUI website. Figure 8A shows the main and the top display once a protein is selected. In Figure 8A, a clickable manual sign on the top left corner toggles the display legend panel. In this panel, numerical values of SSs, N-glycans, unoccupied cysteines, and unoccupied sequons are included. A selection window in the top middle of the display enables the selection of the proteins included in our portal. Currently, it can visualize some extreme cases of PTMs as well as all MPs with conflicts. The middle and main parts of the display show the schematic of the selected protein, in which the polypeptide chain is represented by a bar, and the color of the bar encodes the topology, including the beginning and ending of each TM, and the segment orientation. The positions of N-glycans (the bar) and SSs (colored dots) are specified with unique icons. The linkage of SSs is represented using a bracket, and the same pair has matching color in their dots. Such a display allows users to visualize some complex spatial arrangements of SSs, such as disulfide knots, as shown in Figure 8B. The position of unoccupied sequons is denoted using black dots linked to the letter “N” for the asparagine in the sequon, while the unoccupied cysteines are denoted using white open dots.

We designed two zoom features to accommodate MPs with complex structures. First, an expansion ion “. . . ” on the top right of the display is clickable, which brings out a scaling bar that allows users to customize the scaling. The entire displayed protein will expand accordingly. This often makes the protein too long to be viewed in the window. To compensate for such inconvenience, a particular section of the protein can be selected using the “Protein Window” by specifying the “start” and “end” positions. An expanded view of the disulfide nots in LRP1 is shown in Figure 8B, which is located below the top main window. Rolling the side bar on the right down allows access to this window. The top of this window has two cells that allow the input of a particular region of the selected protein for display. Using the protein visualizer, we displayed the conflicts (also provided in Supplementary Table S5) between the known compartmentalization of SSs and N-GLYCO to the topology depicted by Deep TMHMM and the extreme cases mentioned in the Results and Discussion.

## 3. Materials and Methods

We accessed the Uniprot human reviewed proteome for protein species, annotation, length, SS, and N-GLYCO information. Proteins with either SSs or N-GLYCO or both features were selected for further analysis and were named the SS proteome, N-GLYCO proteome, and SS/N-GLYCO proteome. The FASTA file of the human reviewed proteome was also used for membrane topology prediction using TMHMM 2.0 and Deep TMHMM.

### 3.1. Analysis of General Distribution

We first analyzed the distribution of protein length, counts of SSs, N-glycan, and TMs in the entire human proteome. Second, we obtained the position of cysteine residues and sequons in the SS and N-GLYCO proteomes and evaluated their distribution. We then examined the quantile distribution of SSs, N-glycan, TM, cysteine, and sequons in the SS/N-GLYCO proteome. Specifically, we divided the length of each protein into four equal parts (four quantiles, Q1–Q4) from N-terminus to C-terminus, such that the N-terminus falls in Q1 while C-termini falls in Q4. Finally, we assessed the similarity and uniqueness among the obtained distributions.

### 3.2. Rate of Modification

The rates of sequon occupancy and cysteine utilization were also evaluated in the respective proteomes. These rates were further subjected to correlation analysis for protein length and total number of PTMs per protein as mentioned in the above section.

### 3.3. Hierarchical Clustering

Nonsupervised hierarchical clustering was carried out in Multiple Experiment Viewer (MeV) run on the local machine to the SS/N-GLYCO proteome for the counts of SSs, N-glycans, TMs, cysteine residues, sequons, and the protein length quantile values. A Pearson correlation with the average linkage clustering method was used to generate hierarchical trees of the proteins. Three main clusters were obtained that accounted for all except one protein.

### 3.4. Identification and Analysis of Conflicts

We mapped the predicted topology to each SS, N-glycan, cysteine residue, and sequon in every protein. Using the criteria that SSs and N-glycans are located in the extracellular compartment or in the lumen of the secretory pathway, we identified the conflicts. The proteins on the conflict list were further analyzed for their distribution patterns using the method discussed above for similarities and differences.

### 3.5. Functional Analysis

Special classes of proteins from the abovementioned analyses were assessed for their enriched Gene Ontology terms using the Database for Annotation, Visualization and Integrated Discovery (DAVID) [53]. Specifically, the enrichment in biological processes of Gene Ontology was used to assess their biological functions. The enrichment was determined using the following cutoff parameters: the maximum EASE score (a modified Fisher’s exact *p*-value) of 0.05 and the minimum protein count of 5.

### 3.6. Visualization Tool

We built an interactive web application named “Protein Visualizer” (https://sfu-sun-lab.github.io/protein-visualizer/, accessed on 1 November 2023) using JavaScript (version 1.5) through React.JS framework to display the special relationship between the reported SSs and N-GLYCO to TMs predicted by Deep TMHMM. A total of 8 features are displayed, including protein length, N-GLYCO sites, SS sites, sites of free sequons and cysteines, TMs, and internal and external loops with respect to the plasma membrane, in which the extracellular domain of the protein is depicted in blue, the intracellular domain is represented in pink, and the TM domain is in white. Users can toggle the visibility of all these features. The application also has two built-in scaling features that allow the expansion of the entire protein or a specific section. Currently, the web portal displays 83 conflicting human PMs and a few representative MPs resolved from the above analyses.

## 4. Conclusions

Using the existing PTM information in UniProt on the human proteome and the predicted topology of MPs, we analyzed the hidden relations among TMs, SSs, and N-GLYCO. All three features affect the structure and conformation of MPs and therefore critically influence their functions. All these features have been intensively studied in their own corresponding fields; however, less effort has been focused on their potential relationships. In the past, a serendipitous opportunity led us to discover a complementarity between N-GLYCO and TMs from our experimentally obtained N-glycoproteome [41]. Recently, we further uncovered a complex but closely related interaction between N-GLYCO and SSs in individual proteins by reviewing the published literature [42]. Here, at the human proteome level with the ever-growing accuracy from the computational predictions, we were able to integrate all three structural features together to discover an overall complementarity between TMs and PTMs of SSs and N-GLYCO, in which the TMs do not depend on protein length. On the other hand, the SSs follow an almost identical trend as N-GLYCO in terms of dependence on total protein length, distribution in protein length quantile, and their relationships to TMs, suggesting a similarity between the two PTMs in regulating protein structure and function. With the intriguingly close relationship of enzymes regulating these two PTMs in the ER and the complex relationship of these two PTMs from experimental observations in individual proteins, our analysis at the proteome level suggests that their close relationship not only occurs occasionally in some special proteins but is also more ubiquitously present. Our results encourage more researchers to integrate multiple PTMs and to systematically study proteins carrying them. Furthermore, the tight correlation between sequons and N-GLYCO and consistently less conflict with membrane topology suggest the importance of the sequon position in a protein sequence, which could largely determine the macroheterogeneity of N-GLYCO dynamics. In addition, the wider spread of available cysteine residues and a more constrained presence of sequons from our analysis further hint at a higher dynamic of SSs as opposed to N-GLYCO. Finally, to facilitate future investigation of the hidden relationship between the TM and the two PTMs examined here, we developed a JavaScript-supported “Protein Visualizer” that allows users to visualize these features in detail in individual proteins.

Protein PTMs are an actively studied area. For glycosylation alone, in addition to N-glycosylation, O-glycosylation, in which saccharides are added to the side chains of Ser and Thr, is another common PTM for MPs [54]. However, O-glycosylation is less strict in cellular compartmentalization than N-glycosylation, because O-GlcNAc modification, a special type of O-glycosylation, has been frequently observed in cytosolic and nuclear proteins [55,56]. Interestingly, phosphorylation can compete with O-GlcNAcylation to modify the same side chains of Ser and Thr in certain proteins for cell signaling [57]. How these PTMs contribute to the structure and stability of proteins is another interesting topic that can be further explored in the future. We hope that our efforts will stimulate more studies regarding the correlation between numerous known structural features, and more hidden relationships can be discovered to help better understand functions of MPs and to facilitate future protein engineering and drug design in MPs.

## Data Availability

See Supplementary Materials and our website listed in the text.

## Supplementary Materials

The supporting information can be downloaded at: https://www.mdpi.com/article/10.3390/ijms242216182/s1.
